# Supplementary material for: Preoperative Prediction of New Vertebral Fractures after Vertebral Augmentation with a Radiomics Nomogram
Source: Diagnostics (Basel). 2023 Nov 16;13(22):3459. doi: 10.3390/diagnostics13223459 (PMC10670105; doi:10.3390/diagnostics13223459)
Supplement: Supplementary file 1 [file diagnostics-13-03459-s001.zip › diagnostics-2686231-supplementary.pdf]

Article

Preoperative prediction of new vertebral fractures after vertebral augmentation with a radiomics nomogram

Yang Jiang[1†], Wei Zhang[1†], Shihao Huang[2], Qing Huang[3], Haoyi Ye[4], Yurong Zeng[5], Xin Hua[6], Jinhui Cai[1], Zhifeng Liu[4*], Qingyu Liu[1*]

[1]Department of Radiology, The Seventh Affiliated Hospital, Sun Yat-sen University, Shenzhen 518000, China; jiangy279@mail2.sysu.edu.cn (Y.J.); zhangwei3@sysush.com (W.Z.); caijinhui@sysush.com (J.C.)

[2]Department of Radiology, The Fifth Affiliated Hospital of Sun Yat-sen University, Zhuhai, China 519000; m15285361659@163.com (S.H.)

[3]Department of Endocrinology, The Seventh Affiliated Hospital, Sun Yat-sen University, Shenzhen 518000, China; huangq77@mail2.sysu.edu.cn (Q.H.)

[4]Department of Radiology, The Fourth Affiliated Hospital of Guangzhou Medical University, Guangzhou 510000, China; 2021689031@gzhmu.edu.cn (H.Y.)

[5]Department of Radiology, Huizhou Central People's Hospital, Huizhou 516000, China; zeng_yurong@163.com (Y.Z.)

[6]Department of Neurology, The First Affiliated Hospital of Wenzhou Medical University, Wenzhou 325000, China; huaxin04157915@163.com (X.H.)

[†]These authors contributed equally to this work.

[*] Correspondence: liuqingyu@sysush.com (Q.L.); 2021689182@gzhmu.edu.cn (Z.L.)

1 Supplementary Figures and Tables

1.1 Supplementary Figures

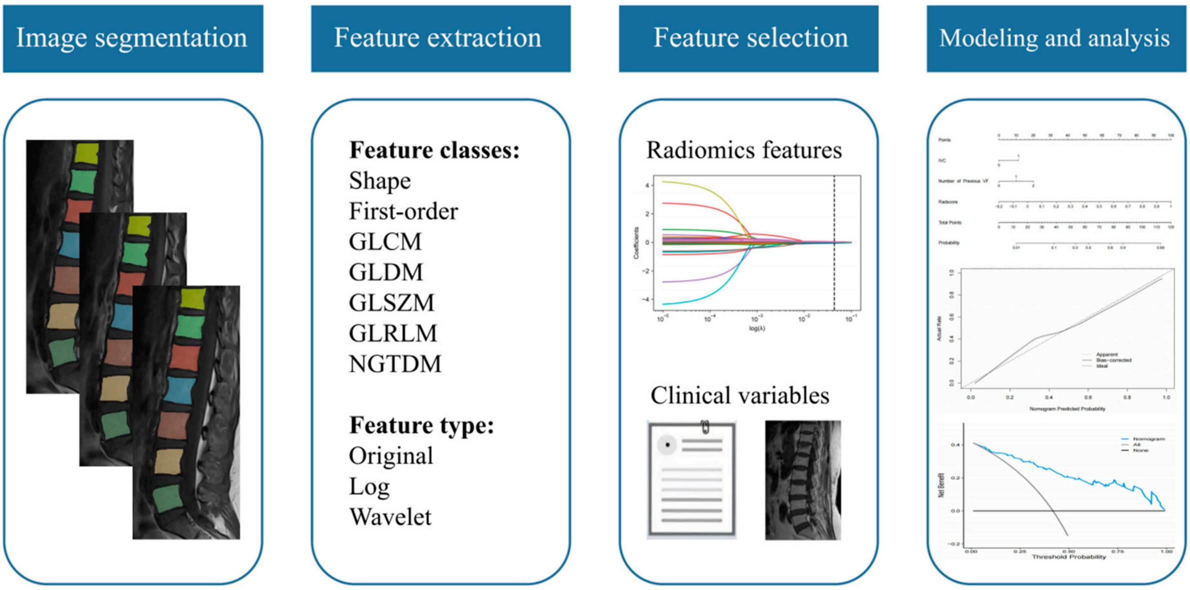

Supplementary Figure S1. The study flowchart and the workflow of radiomics.

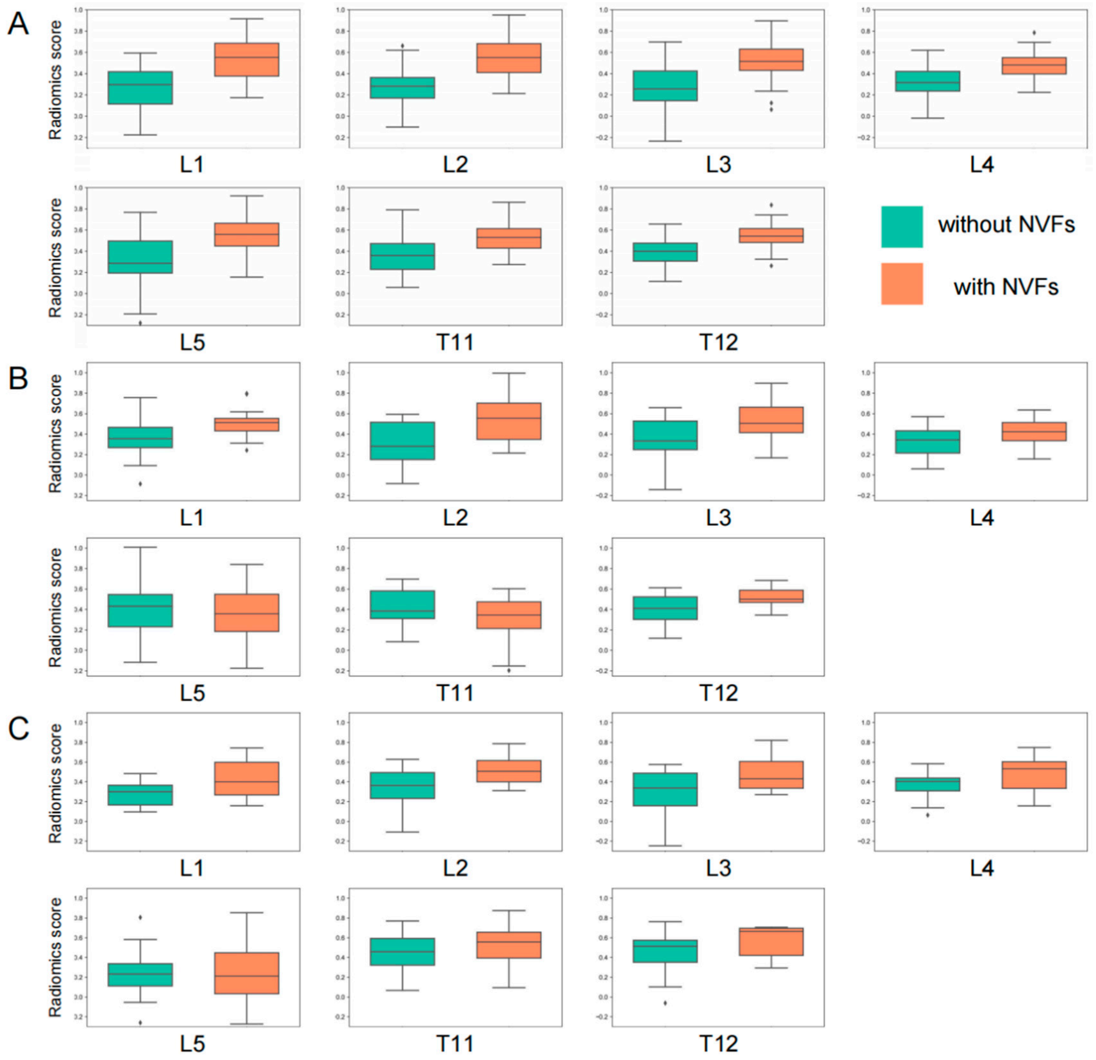

Supplementary Figure S2. The distribution for radiomics scores of each vertebra. (A) training set, (B) internal validation set, (C) external validation set.

1.2 Supplementary Tables

Supplementary Table S1. Details of 1.5 Tesla T1-weighted MRI image acquisition parameters

Parameters	Center 1	Center 2
Scanner	GE (Signa HDi)	Philips (Multiva)
Sequence	TSE	TSE
Repetition / echo time (msec)	450/8.2	384/7.4
Field of view (mm)	300	342
Voxel size(mm)	0.9×1.3×4.0	0.9×1.3×4.0
Bandwidth (Hz)	260	231.5
Thickness(mm)	4	4
Flip angle (degrees)	180	90

Center 1, The Fourth Affiliated Hospital of Guangzhou Medical University; *Center 2*, Huizhou Central People's Hospital.

Supplementary Table S2. Predictive performance of level-specific Radscore compared with clinical variables

	Training set			Internal validation set			External validation set		
Variable	AUC	95%CI	p-value	AUC	95%CI	p-value	AUC	95%CI	p-value
RadscoreT11	0.792	0.714-0.857	<0.001	0.657	0.517-0.780	0.041	0.614	0.445-0.765	0.279
RadscoreT12	0.823	0.736-0.891	<0.001	0.740	0.593-0.856	0.001	0.684	0.478-0.848	0.186
RadscoreL1	0.831	0.740-0.901	<0.001	0.749	0.577-0.878	0.004	0.705	0.481-0.875	0.129
RadscoreL2	0.850	0.773-0.909	<0.001	0.783	0.644-0.887	<0.001	0.751	0.587-0.876	0.002
RadscoreL3	0.825	0.746-0.888	<0.001	0.749	0.608-0.860	<0.001	0.695	0.520-0.837	0.065
RadscoreL4	0.814	0.733-0.879	<0.001	0.686	0.549-0.802	0.015	0.668	0.506-0.806	0.108
RadscoreL5	0.794	0.717-0.857	<0.001	0.574	0.436-0.704	0.371	0.522	0.365-0.677	0.845
Age	0.614	0.532-0.692	0.013	0.602	0.474-0.721	0.144	0.643	0.484-0.781	0.147
Sex	0.539	0.457-0.620	0.309	0.524	0.397-0.648	0.694	0.546	0.379-0.706	0.513
Smoking	0.521	0.439-0.602	0.519	0.524	0.397-0.648	0.636	0.548	0.391-0.699	0.427
BMI	0.533	0.451-0.614	0.486	0.514	0.388-0.639	0.858	0.517	0.362-0.670	0.859
BMD	0.558	0.476-0.638	0.223	0.575	0.447-0.696	0.349	0.559	0.401-0.708	0.527
IVC	0.618	0.536-0.695	0.001	0.610	0.482-0.728	0.032	0.632	0.474-0.772	0.050
Surgical procedure	0.539	0.457-0.620	0.305	0.557	0.429-0.679	0.354	0.522	0.366-0.675	0.766
Number of treated vertebra	0.504	0.422-0.585	0.913	0.594	0.466-0.713	0.107	0.549	0.382-0.709	0.432
Location of treated vertebra	0.570	0.488-0.650	0.073	0.548	0.420-0.671	0.462	0.523	0.357-0.685	0.770
Number of previous VF	0.677	0.597-0.750	<0.001	0.635	0.507-0.750	0.045	0.620	0.461-0.762	0.157

BMD, Bone Mineral Density; BMI,Body Mass Index; CI, confidence interval; IVC, intravertebral cleft; VF, vertebral fracture.

2 Supplementary Data

2.1 Supplementary Appendix S1

RadscoreT11 = -8.6174 + 8.9066×original_glcm_Idmn -

1.7716×log-sigma-3-0-mm-3D_glszm_SmallAreaLowGrayLevelEmphasis +

0.0045×log-sigma-4-0-mm-3D_firstorder_Maximum -

0.4613×log-sigma-4-0-mm-3D_glrlm_ShortRunLowGrayLevelEmphasis +

0.0132×log-sigma-5-0-mm-3D_glrlm_ShortRunHighGrayLevelEmphasis -

0.7246×wavelet-LHH_glszm_SmallAreaLowGrayLevelEmphasis +

0.0062×wavelet-HLL_glszm_HighGrayLevelZoneEmphasis +

0.0948×wavelet-HLL_gldm_DependenceEntropy + 0.8345×wavelet-HHL_firstorder_Median +

0.0738×wavelet-HHH_glcm_SumEntropy -

1.8472×wavelet-HHH_glrlm_RunLengthNonUniformityNormalized +

0.0009×wavelet-HHH_gldm_LargeDependenceHighGrayLevelEmphasis

RadscoreT12 = -0.1503 + 8.8799×original_ngtdm_Strength -

0.8138×log-sigma-5-0-mm-3D_glszm_LowGrayLevelZoneEmphasis +

92.7023×log-sigma-5-0-mm-3D_glszm_ZonePercentage +

0.0037×wavelet-LLH_glszm_GrayLevelNonUniformity +

55.0641×wavelet-LLH_glszm_ZonePercentage -

0.0447×wavelet-LHH_glszm_HighGrayLevelZoneEmphasis +

$7.4154e^{-8}$×wavelet-LHH_glszm_LowGrayLevelZoneEmphasis +

0.1486×wavelet-LHH_gldm_DependenceEntropy -

0.7662×wavelet-HLL_glszm_SizeZoneNonUniformityNormalized +

0.0145×wavelet-HHL_firstorder_Kurtosis -

0.3024×wavelet-LLL_glszm_LowGrayLevelZoneEmphasis

RadscoreL1 = 0.2461 + 0.5674×original_glszm_SmallAreaEmphasis +

0.1498×original_glszm_SmallAreaHighGrayLevelEmphasis + 0.0005×original_ngtdm_Busyness

-29.6950×original_ngtdm_Contrast - 0.1479×log-sigma-3-0-mm-3D_firstorder_Skewness +

1.3256×log-sigma-3-0-mm-3D_glcm_Imc1 + 0.0604×log-sigma-4-0-mm-3D_firstorder_Kurtosis +

0.0435×log-sigma-5-0-mm-3D_firstorder_Kurtosis +

0.0132×wavelet-LLH_glszm_SizeZoneNonUniformityNormalized

-0.0580×wavelet-LLH_glszm_ZoneEntropy - 4.4141×wavelet-LHH_glcm_Imc1 -

0.8285×wavelet-HLL_glszm_SmallAreaEmphasis + 0.0196×wavelet-HLH_firstorder_Kurtosis -

0.0094×wavelet-HLH_firstorder_Minimum

RadscoreL2 = 0.6859 + 0.0048×original_gldm_DependenceVariance +

0.0258×log-sigma-3-0-mm-3D_firstorder_Kurtosis -

0.0996×log-sigma-3-0-mm-3D_glcm_ClusterShade + 1.3215×log-sigma-3-0-mm-3D_glcm_Imc1 +

0.0801×log-sigma-3-0-mm-3D_glrlm_ShortRunHighGrayLevelEmphasis -

0.1367×log-sigma-3-0-mm-3D_glszm_SmallAreaLowGrayLevelEmphasis +

0.4637×wavelet-LHL_glszm_LowGrayLevelZoneEmphasis -

0.0603×wavelet-LHL_glszm_SmallAreaHighGrayLevelEmphasis +

0.0011×wavelet-HLL_glrlm_LongRunHighGrayLevelEmphasis -

0.0446×wavelet-HLH_glszm_GrayLevelNonUniformity -

0.4579×wavelet-HHL_glszm_SmallAreaEmphasis -

0.0724×wavelet-HHL_glszm_SmallAreaLowGrayLevelEmphasis +

0.0768×wavelet-LLL_firstorder_Kurtosis + 0.5691×wavelet-LLL_glcm_Imc1

RadscoreL3 = -1320.7777 + 0.7657×original_glcm_Imc1 +

6.9073e^{-9}×original_glszm_LargeAreaHighGrayLevelEmphasis +

0.0671×log-sigma-4-0-mm-3D_glszm_ZoneEntropy -

0.1525×wavelet-LLH_glszm_SizeZoneNonUniformity +

0.0007×wavelet-LHL_glrlm_LongRunHighGrayLevelEmphasis +

5.4800e^{-5}×wavelet-LHL_gldm_LargeDependenceHighGrayLevelEmphasis +

0.0575×wavelet-LHH_glszm_ZoneEntropy + 0.7547×wavelet-HLL_glcm_Correlation +

0.0379×wavelet-HLL_gldm_DependenceVariance -

19.1784×wavelet-HHL_gldm_DependenceNonUniformityNormalized +

1323.7378×wavelet-HHH_firstorder_Entropy - 2.1655×wavelet-LLL_glcm_Imc2

RadscoreL4 = 5.1828 + 0.0006×log-sigma-4-0-mm-3D_ngtdm_Busyness +

0.0481×wavelet-LHH_glrlm_LongRunLowGrayLevelEmphasis -

0.0848×wavelet-LHH_glszm_SizeZoneNonUniformity -

0.3775×wavelet-HLH_glcm_MaximumProbability -

6.8592×wavelet-HLH_glrlm_HighGrayLevelRunEmphasis -

0.0267×wavelet-HLH_glrlm_ShortRunHighGrayLevelEmphasis +

0.0295×wavelet-HHL_glrlm_LongRunHighGrayLevelEmphasis -

0.0971×wavelet-HHL_glszm_ZoneEntropy + 4.9409×wavelet-HHH_gldm_HighGrayLevelEmphasis

- 0.0005×wavelet-HHH_gldm_LowGrayLevelEmphasis + 1.1897×wavelet-LLL_glcm_Imc1

RadscoreL5 = 67.0056 + 0.3173×wavelet-LLH_glcm_Imc1 -

0.7290×wavelet-LLH_glcm_ClusterTendency + 0.0309×wavelet-LLH_gldm_DependenceVariance +

0.0002×wavelet-LLH_gldm_LargeDependenceEmphasis +

0.0018×wavelet-LLH_gldm_LargeDependenceHighGrayLevelEmphasis +

18.1203×wavelet-LHH_glcm_JointEnergy + 4.9517×wavelet-LHH_glcm_MaximumProbability -

0.6968×wavelet-HLL_glszm_LowGrayLevelZoneEmphasis -

17.9769×wavelet-HLH_glcm_MaximumProbability -

5.7556×wavelet-HHL_glrlm_HighGrayLevelRunEmphasis +

0.0010×wavelet-HHL_glrlm_LowGrayLevelRunEmphasis - 54.7486×wavelet-LLL_glcm_Idmn -

0.1074×wavelet-LLL_glrlm_RunEntropy
